# The lifetime risk of pneumonia in patients with neuromuscular scoliosis at a mean age of 21 years: the role of spinal deformity surgery

**DOI:** 10.1007/s11832-015-0682-8

**Published:** 2015-09-08

**Authors:** Heli Keskinen, Heikki Lukkarinen, Katariina Korhonen, Tuomas Jalanko, Antti Koivusalo, Ilkka Helenius

**Affiliations:** Department of Paediatric Orthopaedic Surgery, Turku University Central Hospital, Kiinamyllynkatu 4-8, 20521 Turku, Finland; Department of Paediatrics, Turku University Central Hospital, Turku, Finland; University of Helsinki, Helsinki, Finland; Hospital for Children and Adolescents, Helsinki University Central Hospital, Helsinki, Finland

**Keywords:** Cerebral palsy, Epilepsy, Neuromuscular, Scoliosis, Pneumonia

## Abstract

**Background:**

Patients with neuromuscular disorders often have an increased risk of pneumonia and decreased lung function, which may further be compromised by scoliosis. Scoliosis surgery may improve pulmonary function in otherwise healthy patients, but no study has evaluated its effect on the risk of pneumonia in patients with neuromuscular scoliosis (NMS).

**Methods:**

The patient charts of 42 patients (mean age 14.6 years) who had undergone surgery for severe NMS (mean scoliosis 86°) were retrospectively reviewed from birth to a mean of 6.1 years (range 2.8–9.5) after scoliosis surgery. The main outcome was radiographically confirmed pneumonia as a primary cause for hospitalization. We excluded postoperative (3 months) pneumonia from the analyses.

**Results:**

The lifetime annual incidence of pneumonia was 8.0/100 before and 13.4/100 after scoliosis surgery (*p* > 0.10). The mean number of hospital days per year due to pneumonia were 0.59 (SD 2.3) before scoliosis surgery and 2.24 (SD 6.9) after surgery (*p* > 0.10). Multivariate analysis demonstrated that lifetime risk factors for pneumonia were epilepsy (RR 15.2, 95 % CI 1.3–176.8, *p* = 0.027), non-cerebral palsy (CP) etiology (RR = 10.2, 95 % CI 3.2–32.7, *p* < 0.001) and major scoliosis (main curve >70°; RR = 11.3, 95 % CI 1.8–70.7, *p* = 0.01).

**Conclusions:**

Epilepsy, non-CP etiology and major scoliosis are significant risk factors for pneumonia in patients with NMS. Scoliosis surgery does not decrease the incidence of pneumonia in patients with severe NMS.

**Level of Evidence:**

Retrospective comparative study, Level III.

**Electronic supplementary material:**

The online version of this article (doi:10.1007/s11832-015-0682-8) contains supplementary material, which is available to authorized users.

## Introduction

Neuromuscular scoliosis (NMS) is caused by a heterogeneous group of neurologic system diseases and neuromuscular disorders like cerebral palsy (CP), myelomeningocele or muscular dystrophy. Patients with neuromuscular disorders typically have restrictive findings in pulmonary function tests due to poor control and function of respiratory muscles [1]. Lung function may be further compromised by progressive scoliosis, which produces lung compression and often a deformed chest cage, thus affecting chest compliance [1]. Pneumonia is one of the most common reasons for acute hospitalization among patients with CP and respiratory failure is the most common cause of mortality [2–4]. Scoliosis in neuromuscular disorders is progressive even after the growth period and severe scoliosis of ≥70 degrees, regardless of etiology, increases mortality because of respiratory failure [5–7].

The effect of neuromuscular scoliosis surgery on pulmonary function has only been studied in patients with progressive muscular dystrophies such as Duchenne and spinal muscular atrophy [7–14]. In adolescent idiopathic scoliosis (AIS), increased spinal deformity is associated with decreased pulmonary function and patients who have undergone posterior spinal fusion with pedicle screw instrumentation have improved pulmonary function after surgery [15–19]. Anterior spinal arthrodesis and thoracotomy may be associated with decreased pulmonary function in AIS [18, 19]. Bracing has been shown to reduce respiratory function in neuromuscular disease [20].

To date, no studies exist on the effect of scoliosis correction on the incidence of pneumonia in patients with NMS. Saito et al. investigated the natural history of 37 untreated CP patients with neuromuscular scoliosis [5]. During the last 3 years of their investigation, seven (19 %) of the 37 patients had pneumonia at least once a year. All patients with curves >40 degrees during adolescence were progressive but the magnitude of scoliosis was not associated with the risk of pneumonia [5].

In this retrospective study we investigated the lifetime risk factors for pneumonia and the effect of scoliosis surgery on the incidence of pneumonia in NMS patients.

## Patients and methods

Forty-two consecutive patients (18 male and 24 female) with NMS living their whole life at Helsinki University Hospital district underwent scoliosis correction surgery at Helsinki University Hospital between 2000 and 2009 (Table 1).

**Table 1:** Characteristics of the study population

	All (*n* = 42)	CP group (*n* = 17)	Non-CP group (*n* = 25)	*p*
Male/female (%)	18 (43)/24 (57)	6 (35)/11(65)	12 (48)/13 (52)	>0.05
Age at surgery (years) (SD)	14.6 (2.6)	15.2 (2,2)	14.1 (2.7)	>0.05
Follow-up time postoperatively (years) (SD)	6.1 (1.7)	6.4 (1.7)	5.9 (1.8)	>0.05
Age at follow-up (years) (SD)	20.6 (3.3)	21.6 (2.4)	20.0 (3.5)	>0.05
Ambulatory (%)	10 (24)	2 (12)	8 (32)	>0.05
Epilepsy (%)	19 (45)	10 (59)	9 (36)	>0.05
Mental retardation (%)	25 (60)	12 (71)	13 (52)	>0.05
Preoperative mean (SD) major curve	86 (20)	93 (20)	81 (19)	0.03
Postoperative mean (SD) major curve	29 (20)	39 (19)	23 (18)	0.004

### Study design

Data on pneumonia and hospitalization were collected retrospectively from medical records and radiographs of the chest and spine. Only pneumonia needing hospital admission was included for analysis. Postoperative (3 months) pneumonia was excluded from the analyses. Inclusion criteria were clinically and radiographically diagnosed pneumonia. The chest radiographs were analyzed by an independent radiologist and the criteria for pneumonia in the radiograph were lobar consolidation, and interstitial or airspace opacities.

We assumed that CP etiology of NMS would be a specific subgroup of patients and we divided the patients into two groups according to diagnosis—patients with CP (*n* = 17) and others (non-CP) (*n* = 25). The non-CP group included one patient with post-traumatic tetraplegia, 17 with syndromic diseases (Table 1 Online supplement), five with myelomeningocele, one with polio and one patient with fetal alcohol syndrome. Of all children, 25 (60 %) had mental retardation and 19 (45 %) had medically treated epilepsy. Retardation was defined according to standardized neuropsychological analysis. Ten of the patients (24 %) were ambulatory. Twenty patients (48 %) had a spinal brace before surgery. Fundoplication was performed in five patients (12 %), and six patients (14 %) received anti-reflux medication before scoliosis correction.

### Scoliosis surgery

The indication for surgery was progressive scoliosis or kyphoscoliosis of ≥60° with poor sitting or standing balance. Twenty-two patients underwent posterior correction surgery only and 20 underwent combined anterior and posterior correction in either one (*n* = 7) or two (*n* = 13) operations. The anterior approach was carried out via thoracotomy/thoracolumbotomy (*n* = 15) or lumbotomy (*n* = 4).

Posterior correction was performed with hybrid instrumentation or total pedicle screw instrumentation [21]. Hybrid instrumentation included bilateral upper thoracic hook claws, sublaminar wires on the concave side and midthoracic hooks on the convex thoracic spine and lumbar pedicle screws. Pedicle screws were inserted free-hand [22]. All patients received prophylactic antibiotics (intravenous cefuroxime) and anti-reflux medication [21].

### Spinal radiographic measurements

Spinal radiographic measurements included proximal thoracic, main thoracic and thoracolumbar/lumbar curves, pelvic obliquity, thoracic kyphosis (T5–T12) and lumbar lordosis (T12–S1) and were performed preoperatively, postoperatively and after follow-up time according to the Cobb method [23, 24]. According to the curve apex location we defined the curve as thoracic (T2–T11/12 disc), thoracolumbar (T12–L1) or lumbar (L1–2 disc through L4) [24].

### Gastroenterological investigations

Gastric emptying was measured preoperatively by imaging the emptying half-life (T_1/2_) of radiolabeled standardized solid (31 patients) and liquid (30 patients) test meals using gamma-ray scintigraphy.

### Statistical methods

The lifetime incidence of pneumonia was analyzed for the total study period and from birth to scoliosis surgery and onwards until the end of follow-up. The risk factors for pneumonia were also analyzed for 3 years both pre- and post-surgery to evaluate more closely the effects of scoliosis surgery on the risk factor characteristics. To estimate the power for comparison of the pre- and postoperative incidence of pneumonia, we performed a priori power analysis using the following parameters—α = 0.05 (type 1 error tolerance); sample size *n* = 35, mean preoperative incidence 20/100, mean postoperative incidence 10/100; standard deviation = 15. We obtained an adequate power of 0.80 (1-β). Data were analyzed using two-way ANOVA, *t*-test, and Mann–Whitney *U* test when appropriate. A negative binomial linear regression model with log link was used for univariable and multivariable risk factor analyses. The logarithmic time-to-event was used as the offset variable. Using the backward stepwise method, the risk factors in the final model were etiology of NMS (CP vs non-CP), epilepsy, and scoliosis >70°. Risk ratio (RR) with 95 % CI was used to express the results.

### Ethical aspects

Ethical committee approval was obtained from the Helsinki and Uusimaa hospital district. No patient contact was needed, so written informed consent was not requested by the Ethical committee.

## Results

The mean age at the time of surgery was 14.6 years (SD 2.6), which was also the mean preoperative follow-up time. The mean postoperative follow-up time to pneumonia was 6.1 years (2.8–9.1 years). During the follow-up time, two (5 %) of the 42 patients died—one due to pneumonia and the other due to non-respiratory reasons. The mean major curve was 86° (SD 20) preoperatively and 32° (SD 20) at the final follow-up. The radiographic data and surgical complications are shown in Online Supplement Tables 2 and 3.

The prevalence and incidence of pneumonia were analyzed for both lifetime and the 3-year period before and after surgery. The prevalence of hospital-treated pneumonia was 36 % (15/42). There were 12 (29 %) patients who had pneumonia before surgery and seven (17 %) who had pneumonia after surgery. Four (9.5 %) patients had pneuomonia in the 3-year period before surgery and six (14 %) patients had pneumonia after the 3-year postoperative period. Furthermore, two patients had pneumonia immediately after scoliosis surgery and these were not included in following results. The annual incidence of pneumonia was 8.0/100 before and 13.4/100 after surgery (*p* > 0.10). The mean number of annual hospital days due to pneumonia were 0.59 (SD 2.3) before and 2.24 (SD 6.9) after surgery (*p* > 0.10).

**Fig. 1:**
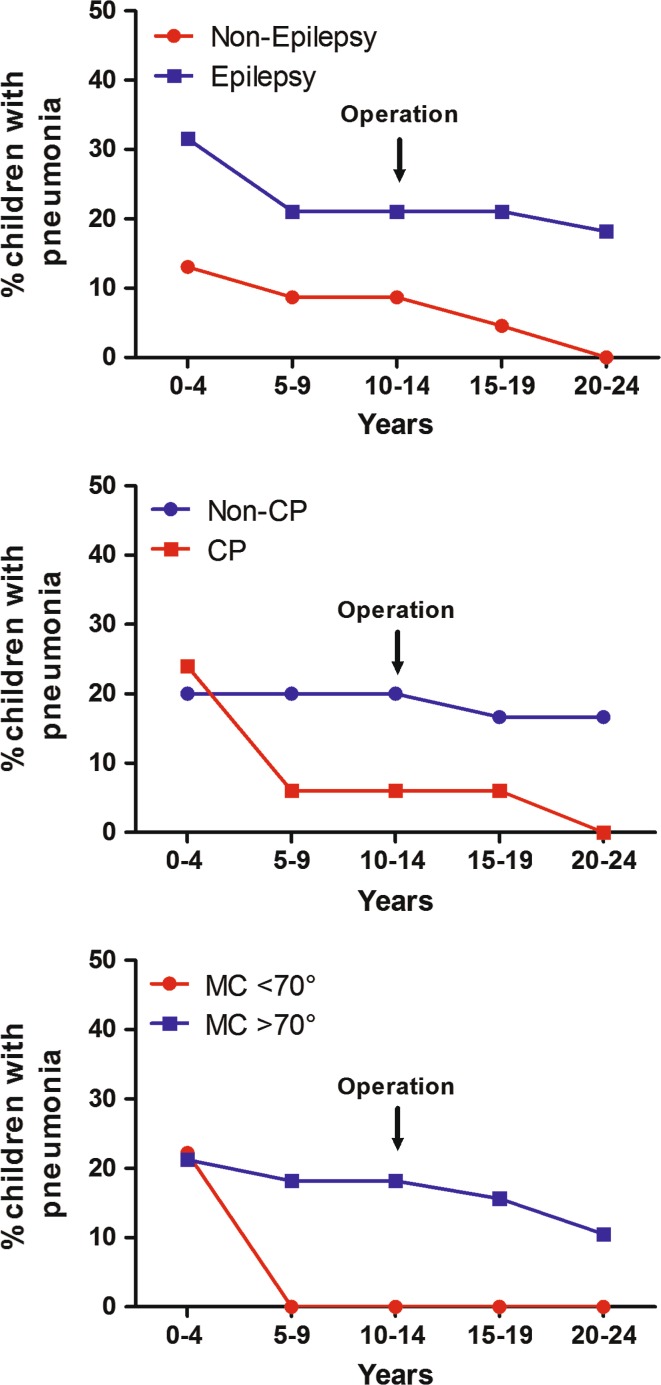
The incidence of pneumonia in children with neuromuscular scoliosis. Epilepsy, non-cerebral palsy (CP) etiology and >70° mean curve (MC) were associated with a higher incidence of pneumonia

Univariable analysis by negative binomial regression demonstrated that the lifetime risk factors for pneumonia during the follow-up were epilepsy, non-CP etiology of the scoliosis, >70° scoliosis before surgery and mental retardation (Table 2). The incidence of pneumonia was 2.0/100 before and 0.7/100 after surgery in CP (*p* = 0.31) and 12/100 and 22/100 in non-CP patients (*p* = 0.13), respectively. However, significant interactions (*p* < 0.001 for both comparisons) were found between epilepsy and non-CP etiology of scoliosis, and epilepsy and retardation. After separating the data according to CP etiology or retardation, epilepsy increased the risk for pneumonia only in children with non-CP-associated scoliosis (RR 11.4, 95 % CI 4.0–32.0, *p* < 0.001) and retardation (RR 24.2, 95 % CI 2.7*–*214.2, *p* = 0.004). The incidence rates of pneumonia in the subgroups are illustrated in the Fig. 1.

**Table 2:** Risk factors for pneumonia in children with neuromuscular scoliosis before scoliosis surgery

Risk factor	Pneumonia			Univariable			Multivariable	
	Incidence/10 years	95 % CI	Risk ratio	95 % CI	*p*	Risk ratio	95 % CI	*p*
Epilepsy								
Yes^a,b^	1.4	0.8–2.5	4.0	1.7–9.7	0.002	8.6	0.8–94.8	0.08
No	0.4	0.2–0.7	1	−	−	1	−	−
Etiology of scoliosis								
Other^b^	1.3	0.8–2.1	5.5	1.9–15.7	0.001	6.7	2.0–21.9	0.002
CP	0.2	0.1–0.6	1	−	−	1	−	−
Preoperative mean curve								
>70°	1.0	0.7–1.6	6.4	1.3–31.5	0.023	6.7	1.1–40.0	0.037
<70°	0.2	0.0–0.8	1	−	−	1	−	−
Gender								
Male	1.1	0.6–1.9	1.5	0.7–3.4	0.32	1.2	0.4–3.2	0.72
Female	0.7	0.4–1.2	1	−	−	1	−	−
Retardation								
Yes^a^	1.1	0.7–1.9	2.6	1.1–6.4	0.040	0.5	0.04–5.8	0.57
No	0.4	0.2–0.9	1	−	−	1	−	−

^a^Epilepsy and retardation had a significant interaction; all retarded patients had epilepsy

^b^Epilepsy and non-CP etiology of scoliosis had a significant interaction

Analysis of the risk factors for pneumonia before and after scoliosis surgery resulted in similar risk factor characteristics (Tables 2, 3) as for total follow-up time (Table 4). Gender did not affect the risk of pneumonia. There was no association between the incidence of pneumonia or hospital days and blood loss or surgery time. Furthermore, there was no increased risk if the patients were using anti-reflux medication, had delayed gastric emptying on scintigraphy or had had fundoplication carried out before scoliosis correction. The surgical technique (anterior or posterior approach) did not have an effect on pneumonia risk.

**Table 3:** Risk factors for pneumonia in children with neuromuscular scoliosis after scoliosis surgery

Risk factor	Pneumonia			Univariable			Multivariable*	
	Incidence/10 years	95 % CI	Risk ratio	95 % CI	*p*	Risk ratio	95 % CI	*p*
Epilepsy								
Yes^a,b^	3.2	1.8–5.6	45.3	5.7–362	<0.001	69.2	7.5–634	0.001
No	0.1	0.0–0.5	1	−	−	1	−	−
Etiology of scoliosis								
Other^b^	2.5	1.5–4.1	27.1	3.4–217	0.002	57	6.1–536	0.016
CP	0.1	0.0–0.7	1	−	−	1	−	−
Preoperative mean curve								
>70°	1.9	1.2–3.0	>100	n/a	<0.001	>100	n/a	<0.001
<70°	0	0.0–0.0	1	−	−	1	−	−
Gender								
Male	1.7	0.9–3.3	1.3	0.5–3.0	0.61	n/a	−	−
Female	1.4	0.8–2.4	1	−	−	1	−	−
Retardation								
Yes^a^	2.4	1.5–4.0	24.4	3.0–195	0.003	n/a	−	−
No	0.1	0.0–0.7	1	−	−	1	−	−

^a^Epilepsy and retardation had a significant interaction; all retarded patients had epilepsy

^b^Epilepsy and non-CP etiology of scoliosis had a significant interaction

**Table 4:** Lifetime risk factors for pneumonia in children with neuromuscular scoliosis at a mean age of 21 years

Risk factor	Pneumonia			Univariable			Multivariable
	Incidence/10 years	95 % CI	Risk ratio	95 % CI	*p*	Risk ratio	95 % CI
Epilepsy							
Yes^a,b^	1.9	1.2–3.2	7.1	3.1–16.6	<0.001	15.2	1.3–176.8
No	0.3	0.1–0.5	1	−	−	1	−
Etiology of scoliosis							
Other^b^	1.6	1.0–2.5	8.4	3.1–22.4	<0.001	10.2	3.2–32.7
CP	0.2	0.1–0.5	1	−	−	1	−
Preoperative mean curve							
>70°	1.3	0.9–1.9	11.5	2.4–56.2	0.003	11.3	1.8–70.7
<70°	0.1	0.0–0.5	1	−	−	1	−
Gender							
Male	1.3	0.7–2.2	1.5	0.7–3.1	0.30	1.2	0.5–3.1
Female	0.9	0.5–1.4	1	−	−	1	−
Retardation							
Yes^a^	1.5	1.0–2.4	4.4	1.9–10.5	0.001	0.5	0.04–5.1
No	0.3	0.2–0.7	1	−	−	1	−

^a^Epilepsy and retardation had a significant interaction, *p* < 0.001

^b^Epilepsy and non-CP etiology of scoliosis had a significant interaction, *p* < 0.001

To further evaluate the effect of spinal deformity surgery, the risk factors for pneumonia during the 3-year periods before and after surgery were examined. The annual incidence rates of pneumonia during the 3-year periods before and after surgery were 4.0/100 and 14.3/100, respectively (*p* > 0.10). Similarly, the number of hospital days in a year because of pneumonia during the 3-year periods before and after surgery were 0.44 (SD 2.0) and 2.56 (SD 10.7), respectively (*p* > 0.10). According to univariable analysis, the significant risk factors for pneumonia during the 3-year follow-up period prior to scoliosis surgery were epilepsy and a preoperative main curve of >70°. However, statistics could not be counted since the patients with no epilepsy or major scoliosis had no pneumonia events during the 3-year period prior to surgery. Three years after scoliosis surgery the risk factors were epilepsy (RR 21.8, 95 % CI 2.9–163, *p* = 0.003), non-CP etiology (RR 12.2, 95 % CI 1.6–91.7, *p* = 0.015), and a preoperative main curve of >70° (RR > 100, *p* < 0.001).

## Discussion

An improvement in lung function and a potential decrease in the incidence of pneumonia have been the major goals for scoliosis surgery in patients with neuromuscular disease. However, little is known about the natural history of pulmonary function impairment as well as the incidence of pneumonia.

In this study, we provide evidence that there are specific subgroups of NMS patients that may be more susceptible for pneumonia. Furthermore, late scoliosis surgery (at the time the main curve is >70°) does not decrease the incidence of pneumonia in patients with NMS of various origins.

Severe scoliosis of ≥70 degrees has been shown to increase mortality compared with the normal population [6]. In patients with AIS, restrictive lung disease does not usually occur before curves of >60 degrees [16]. In these patients, small increments in lung function can be obtained after posterior spinal surgery [17, 18]. In patients with neurologic conditions and associated scoliosis, i.e., neuromuscular scoliosis, the effects on lung function are much less studied, since normal lung function testing is not usually possible, except in patients with muscle dystrophies such as Duchenne muscular dystrophy [7–14]. In the current study, we observed that the incidence of hospital-treated pneumonia was strongly associated with epilepsy, non-CP etiology and severe scoliosis. Scoliosis surgery did not decrease the incidence of pneumonia in patients with neuromuscular scoliosis; however, on the contrary, there was a trend towards an increased incidence of pneumonia in non-CP patients.

### Validity of the data

Our main outcome, hospital-treated pneumonia, was based on both clinical and radiographic findings and is therefore comparable between patients. Data on pneumonia and days spent in hospital were complete. The chest radiography findings confirmed the diagnosis of pneumonia; however, we may have missed some cases because not all radiographs show findings of pneumonia. Hospital-treated pneumonia was selected as the main outcome instead of all respiratory tract infections to increase the reliability and clinical relevance of the condition. Multivariate analyses were used to evaluate the risk factors for pneumonia. The weakness of our study is the lack of a control group; however, according to the recommendations, it would be unethical to follow patients with progressive neuromuscular scoliosis without surgery. It is possible that some non-CP patients had progressive neurologic disease, which could contribute to decreased lung function and an increased risk of pneumonia; however, none of the patients in this series had progressive muscular dystrophy such as Duchenne or spinal muscular atrophy, which would clearly support this bias. In contrast, even patients with static neurologic conditions such as CP did not demonstrate a decreased incidence of pneumonia after spinal deformity surgery. We were not able to show any difference in the risk of postoperative pneumonia between the posterior only approach and the combined approach; however, the study groups were relatively small especially if CP and non-CP patients were analyzed separately.

### Comparison with previous data

The effect of scoliosis surgery on the risk of pneumonia has been unclear as no study has evaluated the overall risk of pneumonia in these patients. It has been shown that patients with NMS benefit from scoliosis correction because of better quality of life after surgery, improved activities of daily living and care given, better sitting balance and correction of spinal deformity [14, 25, 26]. Few studies exist on the effect of scoliosis surgery on pulmonary function in children with NMS. Most of the studies have been performed in patients with muscular dystrophies, i.e., Duchenne muscular dystrophy [7–14]. Findings are somewhat controversial—some authors suggest that pulmonary function continues to decline after scoliosis surgery but the rate of decline is decreased, while others have reported no improvement in pulmonary function after surgery compared to preoperative or non-operative rate of forced vital capacity (FVC) decline [7, 8, 10–14]. Outcomes of correlation between pulmonary dysfunction level and the severity of scoliosis are also unclear [9, 10]. FVC has been used in previous studies to investigate the effect of scoliosis correction on pulmonary function in NMS patients [7–14]. In our experience the most severe patients with NMS are not able to perform reliable tests with a spirometer because of poor cooperation. Furthermore, most of the lung function studies have been performed among Duchenne muscular dystrophy patients and these results should therefore not be generalized to all neuromuscular patients [7, 9–11, 13, 14].

In our study population, the incidence of pneumonia during the first years of life was higher than later in life and then the incidence declined, which is similar to that seen in the normal population (see Fig. 1) [27]. As there were four patients who had pneumonia before and after surgery, we were not able to show any changes in the number of cases of pneumonia even in individual patients before and after surgery. Additionally, the mean number of annual hospital days increased from the preoperative to the postoperative follow-up period. Unexpectedly, gastroesophageal reflux did not increase the risk for pneumonia according to this study, as there was no association between pneumonia and patients having anti-reflux medication or fundoplication surgery. Furthermore, delayed gastric emptying, which could increase the risk for aspiration, did not increase the risk for pneumonia. The gastric emptying test was performed only before scoliosis correction, but previous studies have shown that scoliosis correction does not significantly affect the gastric emptying time [28]. In neurologically impaired patients who are mentally retarded, the risk for aspiration pneumonia has been proposed to be increased because of a combination of factors such as increased oral secretions, impaired swallowing and epilepsy [29]. In our study, epilepsy increased the risk for hospital-treated pneumonia only in children with non-CP-associated scoliosis and retardation. Anti-epileptic medications may have an immunosuppressive effect that may predispose NMS patients to bacterial infections [30]. Overall, the mechanisms for increased pneumonia risk are multifaceted and no single cause for the risk can be determined.

We hypothesize that severe NMS (mean 86° in this patient group) may produce an irreversible reduced lung function even in patients with static neurologic conditions, such as CP, and therefore it may not be improved by spinal deformity correction. The natural history of neuromuscular scoliosis, e.g., in patients with CP, has been shown to be progressive >40° [5]. In the current study, a preoperative major curve of ≥70° was associated with a significant risk of pneumonia. Therefore, the question remains as to whether we should re-consider our current indication (≥60°) as appropriate [31]. Should we operate on patients with neuromuscular scoliosis before it contributes to a further reduction in lung function in these patients?

## Conclusions

NMS is associated with an elevated risk for pneumonia. Medically treated epilepsy, non-CP etiology and preoperative scoliosis of >70° increase the risk for pneumonia. Scoliosis surgery does not decrease the incidence of pneumonia in patients with severe NMS.

## Electronic supplementary material

Supplementary material 1 (DOCX 50 kb)
