# Supplementary material for: The lifetime risk of pneumonia in patients with neuromuscular scoliosis at a mean age of 21 years: the role of spinal deformity surgery
Source: J Child Orthop. 2015 Sep 8;9(5):357–64. doi: 10.1007/s11832-015-0682-8 (PMC4619373; doi:10.1007/s11832-015-0682-8)
Supplement: Supplementary file 1 — Supplementary material 1 (DOCX 50 kb) [file 11832_2015_682_MOESM1_ESM.docx]

**Online supplement Table 1.**

Diagnoses of 17 patients with syndromic disease.

| **Dianosis** | **n** |
| --- | --- |
| **Chromosomal disorder (45Y)** | 1 |
| **Prader-Willi** |  |
| **Unknown disorder**  **Charcot Marie Tooth** | 6  2 |
| **Cri du Chat** |  |
| **Rett syndrome** |  |
| **Microcephalia** |  |
| **Xanthogranuloma juvenilis** |  |
| **Leigh's syndrome** |  |
| **Cortical dysplasia** |  |
| **Miller-Dieker syndrome** |  |

**Online Supplement Table 2.**

Radiographic results.

|  | **All**  **(n=42)** | **CP**  **(n=17)** | **Other diagnosis**  **(n=25)** | **P** |
| --- | --- | --- | --- | --- |
| **Mean (SD) major curve (°)**  **Preoperative**  **Correction on traction**  **Postoperative**  **Final follow-up** | 86 (20)  37% (17)  29 (20)  32 (20) | 93 (20)  34% (16)  39 (19)  40 (16) | 81 (19)  39% (17)  23 (18)  26 (21) | P=0.03  p>0,05  p=0.004  p=0.01 |
| **Mean (SD) T5-T12 kyphosis (°)**  **Preoperative**  **Postoperative**  **Final follow-up** | 47 (22)  34 (13)  37 (18) | 46 (26)  32 (14)  38 (16) | 48 (19)  35 (12)  37 (19) | p>0,05  p>0,05  p>0,05 |
|  |  |  |  |  |
| **Mean (SD) T12-S1 lordosis (°)**  **Preoperative**  **Postoperative**  **Final follow-up** | 51 (16)  47 (12)  49 (17) | 54 (15)  49 (14)  51 (14) | 49 (16)  47 (11)  47 (19) | p>0,05  p>0,05  p>0,05 |
| **Mean (SD) sagittal balance (mm)**  **Preoperative**  **Final follow-up** | 55 (45)  51 (43) | 32 (53)  32 (36) | 70 (32)  60 (44) | p=0.006  p=0.04 |
|  |  |  |  |  |
| **Mean (SD) pelvic obliquity (°)**  **Preoperative**  **Final follow-up** | -1 (23)  0,15 (11) | -5 (25)  -1 (13) | 3 (21)  1 (9) | p>0,05  p>0,05 |
|  |  |  |  |  |
| **Mean (SD) coronal balance (mm)**  **Preoperative**  **Final follow-up** | -15 (45)  -4 (32) | -23 (53)  -14 (40) | -10 (40)  2 (26) | p>0,05  p>0,05 |
|  |  |  |  |  |

**Online Supplement Table 3.**

Intraoperative and postoperative complications. 10 (10/42) patients had intraoperative complications and 19 patients had one or more postoperative complications. There were no hemo- or pneumothorax complications.

|  | **n** |
| --- | --- |
| **Intraoperaoperative complications** |  |
| Dural lesion | 4 |
| Perforation of pleura | 3 |
| Perforation of peritoneum | 3 |
| Anaphylactic reaction | 1 |
| **Postoperative complications** |  |
| Implant failure | 5 |
| Paralytic ileus | 4 |
| Deep wound infection | 4 |
| Transient paraparesis | 1 |
| Septic infection in urinary tract | 1 |
| SIADH* | 1 |
| Unclear infection | 2 |
| Pneumonia | 2 |
| Pyelonephritis | 1 |
| Transient neurologic defect in lower limb | 1 |
| Neuropathic pain in thoracolumbar scar | 1 |

***** syndrome of inappropriate antidiuretic hormone hypersecretion
